# The possible route of introduction of bluetongue virus serotype 3 into Sicily by windborne transportation of infected *Culicoides* spp.

**DOI:** 10.1111/tbed.13201

**Published:** 2019-04-29

**Authors:** Cecilia Aguilar‐Vega, Eduardo Fernández‐Carrión, José M. Sánchez‐Vizcaíno

**Affiliations:** ^1^ VISAVET Health Surveillance Centre Universidad Complutense Madrid Madrid Spain; ^2^ Animal Health Department, Faculty of Veterinary Medicine Universidad Complutense Madrid Madrid Spain

**Keywords:** bluetongue, *Culicoides*, introduction, serotype 3, windborne

## Abstract

In October 2017, the first outbreak of bluetongue virus serotype 3 (BTV‐3) began in Italy, specifically in western Sicily. The route of entrance remains unclear, although since 2016 the same strain had been circulating only 150 km away, on the Tunisian peninsula of Cape Bon. The present analysis assessed the feasibility that wind could have carried BTV‐3‐infected *Culicoides* spp. from Tunisia to Sicily. An advection‐deposition‐survival (ADS) model was used to estimate when and where *Culicoides* spp. were likely to be introduced prior to the first BTV‐3 report in Italy. Additionally, the Hybrid Single‐Particle Lagrangian Integrated Trajectory (HYSPLIT) model was used to support ADS outputs. The modelling suggests that during September 2017, strong wind currents and suitable climatic conditions could have allowed the transportation of *Culicoides* spp. from BTV‐3‐infected areas in Tunisia into Sicily. ADS simulations suggest that particles could have reached the province of Trapani in western Sicily on 2 and 12 September. These simulations suggest the feasibility of aerial transportation of infected *Culicoides* spp. from Tunisia into Sicily. They demonstrate the suitability of the ADS model for retrospective studies of long‐range transportation of insects across large water bodies, which may enhance the early detection of vectorial disease introduction in a region.

## 1 INTRODUCTION

Bluetongue (BT) is an infectious, non‐contagious, arboviral disease listed by the World Organisation of Animal Health (OIE). It affects primarily ruminants and is caused by bluetongue virus (BTV), which belongs to the family Reoviridae genus *Orbivirus* (Verwoerd & Erasmus, 2004). BT has been reported on all habitable continents (Verwoerd & Erasmus, 2004), and its distribution is linked to that of its vector, small biting midges of the genus *Culicoides* (Diptera: Ceratopogonidae) (Purse, Brown, Harrup, Mertens, & Rogers, 2008). Some *Culicoides* species are biological vectors not only of BTV but also of pathogens that cause other diseases of veterinary importance (Mellor, Boorman, & Baylis, 2000).

Bluetongue, like other vector‐borne diseases, can be introduced into new regions through legal and illegal movements of susceptible hosts or through transportation of the vector in vehicles, on the wind, in semen and in embryos (Mintiens et al., 2008). One worrying and uncontrollable route of BT introduction into southern Europe is the windborne advection of infected *Culicoides* spp. from BT‐affected regions in North Africa. *Culicoides* midges can be transported long distances (Reynolds, Chapman, & Harrington, 2006), even as far as 700 km under certain climatologic conditions when wind speed is sufficient (Sellers, Pedgley, & Tucker, 1977) and orographic barriers are absent (Bishop, Spohr, & Barchia, 2004; Hendrickx et al., 2008).

**Figure 1:**
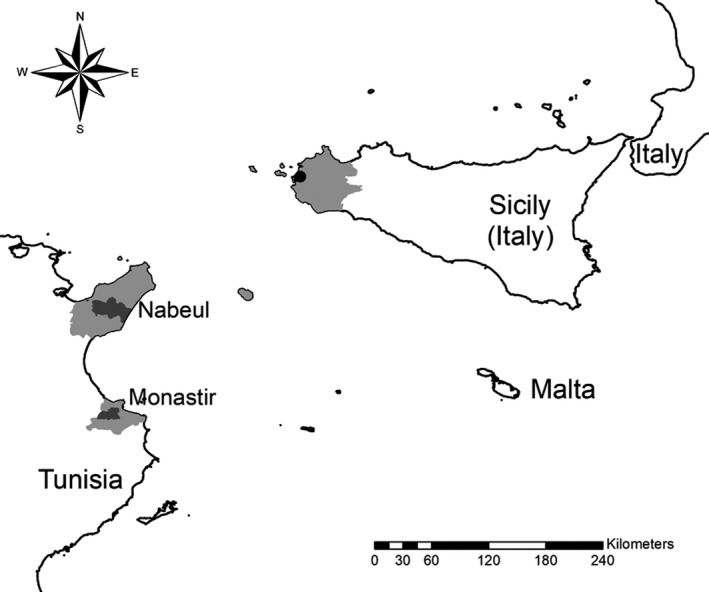
Map showing areas affected by BTV‐3 TUN2016 in Tunisia and Sicily. Light grey areas show the administrative areas affected in Tunisia and Sicily. Dark grey areas show the affected Tunisian Delegations where BTV‐3 TUN2016 were detected in Nabeul and Monastir Governorates (Lorusso et al., 2018). The Sicilian outbreak of BTV‐3 is marked with a black circle

In November 2016, a BT outbreak was identified in the Beni Khalled delegation in the Nabeul Governorate in north‐eastern Tunisia (OIE, 2017a) (Figure 1). The causative agent was identified as a new strain of BT serotype 3 (BTV‐3) called BTV‐3 TUN2016 (Sghaier et al., 2017). This was the first time BTV‐3 had ever been reported in the country. BTV‐3 RNA and antibodies were found to be widespread in Tunisia at the end of 2016 and beginning of 2017, and a different strain of BTV‐3 was detected farther south (Lorusso et al., 2018). In December 2017, Italian authorities reported to the OIE an outbreak of BTV‐3 beginning on 26 October in the Trapani municipality in western Sicily (OIE, 2017b) (Figure 1). This serotype was not known to be circulating in Italy or any other European country. One crossbred sheep (*Ovis aries*) in a flock of 443, presented BT‐like clinical signs (fever, head oedema, nasal discharge and depression). Samples from the affected sheep were tested at the Istituto Zooprofilattico Sperimentale of Sicily, and found to be positive for BTV RNA and anti‐BTV antibodies (Lorusso et al., 2017). Samples were sent to the Italian National Reference Laboratory for confirmation and strain identification, where a seroneutralization test with all reference BTV serotypes gave a positive result for BTV‐3 (Lorusso et al., 2017) on 24 November 2017 (OIE, 2017b). Furthermore, preliminary sequencing analysis demonstrated high nucleotide identity with strain BTV‐3 TUN2016 circulating in northern Tunisia (Lorusso et al., 2017). Fewer than 150 km separate the western coast of Sicily and the eastern coast of Cape Bon peninsula, where the Nabeul Governorate is located. This proximity suggests the possibility of windborne BTV‐3 introduction from Tunisia to Sicily.

To determine the feasibility of windborne introduction, this study applied two atmospheric dispersion models to examine long‐range aerial transportation of infected *Culicoides* spp. Recently, our group developed an advection‐deposition‐survival (ADS) model to assess the risk of introduction of engorged and potentially infected midges in Spain (Fernández‐Carrión et al., 2018). This model is updated hourly, making it a potentially powerful tool for surveillance. In parallel, we analysed data using the Hybrid Single‐Particle Lagrangian Integrated Trajectory (HYSPLIT) model (version 4) of the Air Resources Laboratory at the US National Oceanic and Atmospheric Administration (Draxler & Hess, 1998). Our analyses suggest that the ADS model can be used in retrospective studies of disease vector transportation across large water bodies and, therefore, as a potential early warning tool.

## 2 MATERIALS AND METHODS

### 2.1 Analysis of windborne *Culicoides* trajectories and ground deposition

In order to determine whether windborne advection is a feasible route of introduction of BTV‐3 in western Sicily, continuous simulations were carried out with ADS and HYSPLIT models to identify possible days and times of day when introduction occurred.

The ADS model was developed for estimating risk of wind‐borne introduction of flying insects into a country. It identifies geographic areas and periods where and when risk of vector‐borne diseases incursion is high. Concretely, the model predicts the number density of introduced small flying insects such as *Culicoides* spp. over space and time based on wind advection, vertical deposition rate and insect survival rate (Fernández‐Carrión et al., 2018). The model assumes an initial number density of midges in the source territory (in this case, the Cape Bon peninsula in Tunisia), based on the *Culicoides* spp. activity function, which correlates with local temperature. Then the model simulates the long‐range transportation of midges using Lagrangian particle tracking based on wind direction and intensity until their deposition in the target territory (in this case, Italy and Malta). The model takes into account specific physical and aerodynamic properties of the transported midges (i.e., midge density, Reynolds number and drag coefficient). It also computes a survival rate, which depends on temperature and reflects the probability that an insect may survive transportation at high altitudes, where temperatures can be extreme (Fernández‐Carrión et al., 2018). A modification was implemented for the estimation of Culicoides' density (*ρ* = *m*/*V*), for which midge volume (*V*) was estimated according to the formula: ln(*V*) = 1.019ln(*m*_dry_) + 1.46, where *m*_dry_ refers to dry midge weight (Kühsel, Brückner, Schmelzle, Heethoff, & Blüthgen, 2017), which in turn was estimated to be 30% of total weight (Wigglesworth, 1972). Hourly mean temperature (in °C), mean horizontal wind direction and wind speed (in m/s) were retrieved from an online climatic repository (http://www.forecast.io) for the area and the study period, at temporal resolution of 1 hr (Fernández‐Carrión et al., 2018).

The HYSPLIT model (available online at https://ready.arl.noaa.gov/) is a complex atmospheric system for computing simple air particle trajectories as well as complex transport, dispersion and deposition simulations using archived meteorological data (Stein et al., 2015). The HYSPLIT model is widely used in atmospheric sciences (Stein et al., 2015), and it has even been used to study windborne transportation of* Culicoides* spp. (Durr, Graham, & van Klinken, 2017; Eagles, Walker, Zalucki, & Durr, 2013; García‐Lastra et al., 2012), even though their physical and aerodynamic features differ substantially from those of sand or aerosols. The model relies on meteorological data obtained through the Real‐time Environmental Applications and Display System and archived in the Global Data Analysis System (GDAS1), with temporal resolution of 3 hr.

In both models, the initial altitude and initial positions of particles can be specified. More detailed information is available elsewhere about the methodology and specifications of the ADS model (Fernández‐Carrión et al., 2018) and HYSPLIT model (Draxler & Hess, 1998; Stein et al., 2015).

### 2.2 Study period and area

**Figure 2:**
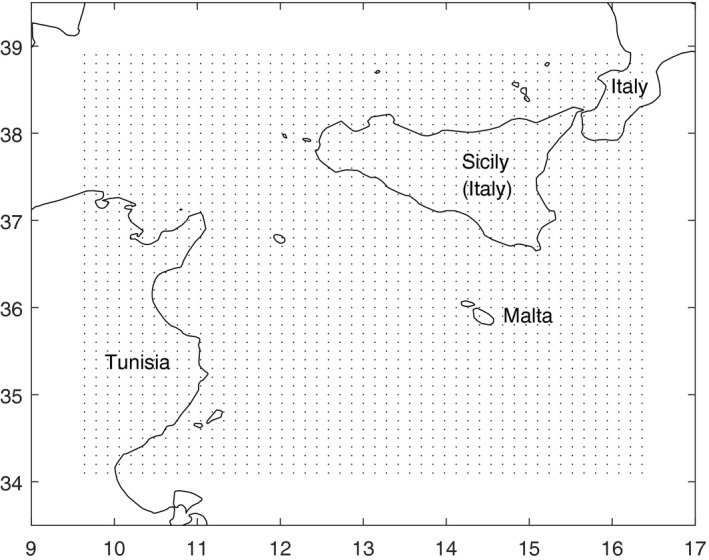
Study domain between latitudes 34ºN and 39ºN, and between longitudes 9.5ºE and 16.5ºE. Dots represent the center of grid cells of 49 x 49 (2,401 in total) used for climatic data interpolation. Grid cell size is 0.1 x 0.14°

BT serological response appears between 7 and 14 days post‐infection (OIE, 2014). Since the BTV‐3 outbreak in Sicily started on 26 October 2017 (OIE, 2017a), our modelling focused on the period from 1 August to 18 October 2017. To configure the ADS model, we set the extension of the study area to lie between latitudes 34ºN and 39ºN and between longitudes 9.5ºE and 16.5ºE. This area includes north‐eastern Tunisia, Sicily, Malta, and the south‐western area of mainland Italy (Figure 2). Within the study area, a grid of 49 x 49 points was created for retrieval of meteorological data. In the ADS and HYSPLIT models, the source of potentially infected midges was set as the Cape Bon peninsula (Tunisia), since BTV‐3 was known to be in that area (Lorusso et al., 2018). The area of potential introduction was mainland Italy, the Italian islands and Malta (which fell within the study area).

### 2.3 Model simulations

A simulation was performed with the ADS model at 1,000 metres above sea level for the study period in order to identify the days and times of highest probability of midge introduction in Sicily from the Cape Bon peninsula. Table 1 shows the major physical and aerodynamic properties of the midges. Once the potential days of introduction were determined, they were studied in greater detail using both the ADS and HYSPLIT models. In the HYSPLIT model, forward concentrations from the Cape Bon peninsula were calculated for time intervals of up to 12 hr at 900 metres above ground level. The maximum flight duration used in this study for the HYSPLIT model agrees with midge survival estimates from other studies (Agren, Burgin, Lewerin, Gloster, & Elvander, 2010; Durr et al., 2017; Eagles, Deveson, Walker, Zalucki, & Durr, 2012). HYSPLIT concentration analysis was performed with 1,000 inert particles of the same mass and density as in the ADS model and a dry deposition velocity of 0.005 m/s (Agren et al., 2010; Durr et al., 2017); particles were released for a period of 3 hr from the source region. Account was taken of the prevailing winds coming into Sicily on the prospective days of midge introduction based on wind roses at coordinates of the Sicilian outbreak obtained from the Air Resources Laboratory at the US National Oceanic and Atmospheric Administration (https://ready.arl.noaa.gov/).

**Table 1:** Parameters included in the ADS model

Parameter acronym	Parameter description	Value in the study	Value reference
Rc	Culicoides' radius (m)	1.01 · 10^−3^	(Morag et al., 2013)
Mc	Culicoides' weight (kg)	5.77 · 10^−7^	(Leprince et al., 1989)
*ρ*	Culicoides' density (kg/m^3^)	800.34	Derived from (Kühsel et al., 2017)
*α*_1_	Hellmann exponent above open water surfaces	0.10	(Fernández‐Carrión et al., 2018)
*α*_2_	Hellmann exponent above land surfaces	0.30	(Patel, 2006)
Re	Culicoides' Reynolds number	120.00	(Shyy & Liu, 2007)

## 3 RESULTS

### 3.1 Model outputs

**Figure 3:**
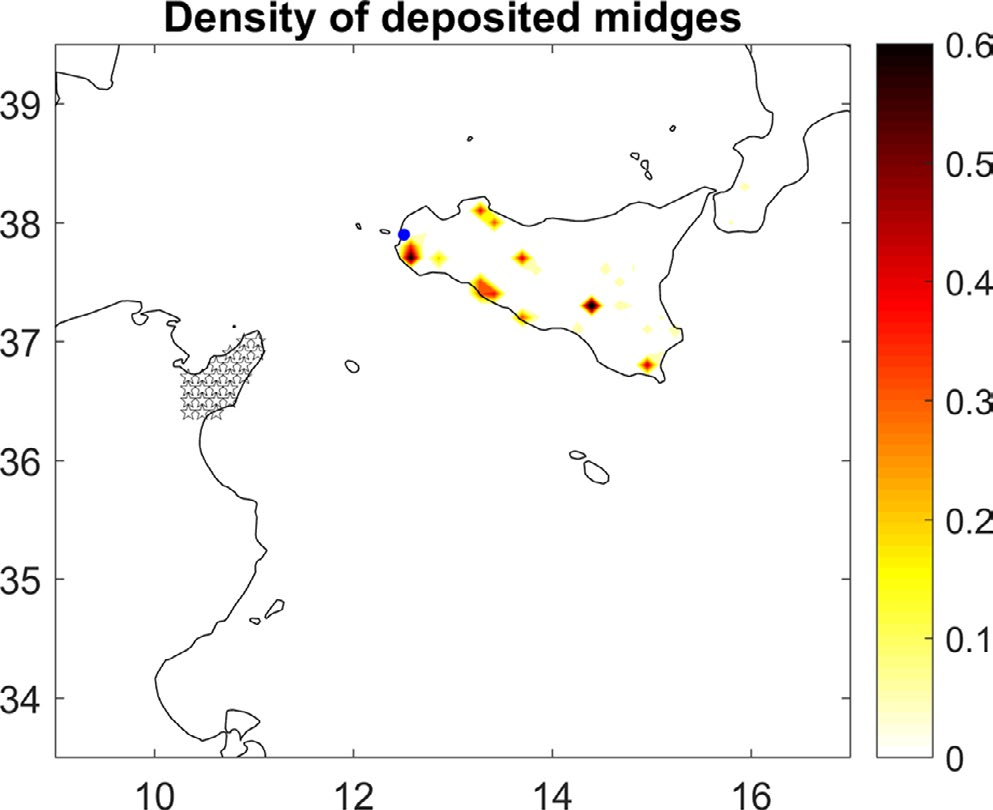
Number density of deposited midges in the target territory for the study period (1 August–18 October, 2017) according to the ADS model. The blue dot shows the location of the Sicilian outbreak of BTV‐3. Stars represent the source of midges in the Cape Bon peninsula [Colour figure can be viewed at http://www.wileyonlinelibrary.com]

Figure 3 shows the density of particles deposited in Sicily during the study period in the ADS simulation. Slight incursions of particles were predicted to scatter along Sicily, with main deposits on the southern coast and the south‐western area of the island. In August, no particle was predicted to land in Sicily. In September, only 4 days of potential arrival of infected midges to target territory were predicted: days 2, 10, 11, and 12. In October, deposition was predicted only on day 6. The most probable times of midge incursion and deposition in Sicily on these five dates were predicted using both models (Table 2). No particles were predicted to reach Malta during the study period.

**Table 2:** Days and time period of deposition of *Culicoides* spp. in the target territory according to the ADS model at 1,000 m above sea level and the HYSPLIT model at 900 m above ground level

Day and month of midge/particle deposition	ADS model						HYSPLIT model					
	Mean number density of midges at source, log(*C* + 1)^a^	Hour of deposition (UTC + 1)	Number density of deposited midges, log(*C* + 1)		Percentage of number density of deposited midges (%)		Time of release (UTC + 1)	Time of simulation end (UTC + 1)	Deposited particles/km^2^		Percentage of deposited particles (%)	
			Sicily	Trapani	Sicily	Trapani			Sicily	Trapani	Sicily	Trapani
02 September	4.10	11 hr	1.06	1.06	16.25	79.21	5 hr	11 hr	8,028.71	624.56	35.14	43.37
10 September	4.65	21 hr	0.46	0	7.06	0	11 hr	23 hr	14,703.80	806.16	64.36	55.98
11 September	4.01	7–14 hr	3.88	0.05	59.41	3.77	8 hr	16 hr	0	0	0	0
12 September	4.69	2 hr	0.23	0.23	3.49	17.01	7 hr	19 hr	113.66	9.46	0.50	0.66
06 October	3.70	19 hr	0.90	0	13.80	0	11 hr	21 hr	0	0	0	0

a based on the activity function of the ADS model.

**Figure 4:**
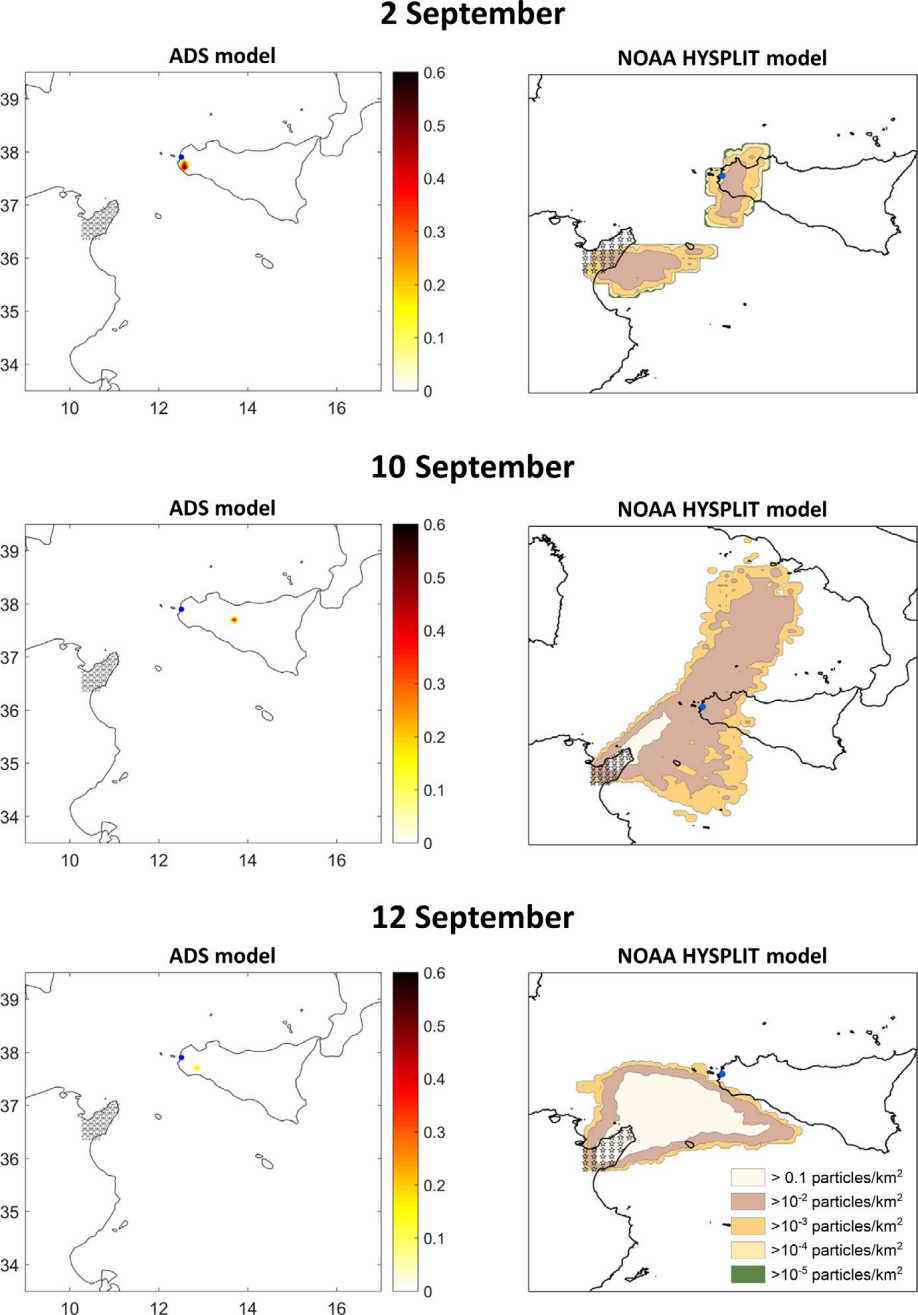
Particle deposition according to ADS and HYSPLIT models for the potential days of introduction. The blue dot shows the location of the Sicilian outbreak of BTV‐3. Stars represent the source of midges and particles in the Cape Bon peninsula. Deposition time of the ADS model and the initial and final time of HYSPLIT outputs are gathered in Table 2 [Colour figure can be viewed at http://www.wileyonlinelibrary.com]

Figure 4 shows the density of particles deposited in the target territory on the days when either model predicted that particles were deposited in western Sicily. On 2 September, Trapani province was predicted by both models to accumulate most of the deposition in the study period. On 10 September, the HYSPLIT model predicted deposition of particles in the Trapani region, in contrast to the ADS model. Both models predicted that on 12 September, there was scarce deposition in Trapani. The ADS model predicted that deposition occurred in south‐central and eastern Sicily on 11 September and again in eastern Sicily on 6 October. The HYSPLIT model, in contrast, predicted no deposition on these dates. Thus, these dates were not considered as potential days of introduction in further analyses.

**Figure 5:**
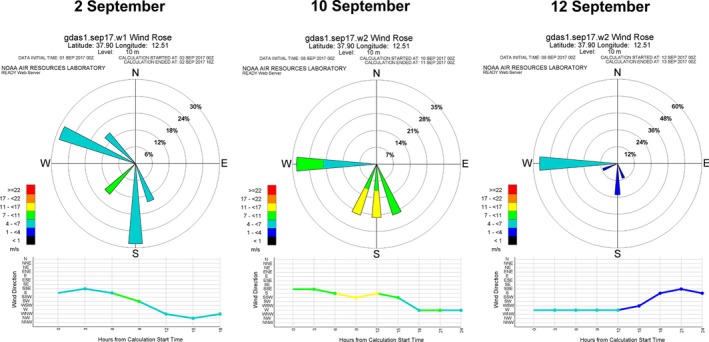
Wind roses show wind speed and direction in the Italian outbreak location at ground level on the potential days of midge introduction. Wind rose diagrams show wind direction (from where the wind comes), its frequency and the wind velocity identified with different colours. Graphics show direction and intensity of the wind at the temporal resolution of the GDAS1 meteorological data [Colour figure can be viewed at http://www.wileyonlinelibrary.com]

During the dates considered to be at high risk of vector‐borne introduction (2, 10, and 12 September), wind directions were predicted to enhance the arrival of midges to the location of the outbreak in Sicily (Figure 5). On 2 and 12 September, wind speeds at ground level were moderate to low during the entire day, ranging from four to less than 11 m/s on 2 September and, from one to less than 7 m/s on 12 September. On 10 September, winds were stronger and reached speeds of up to 17 m/s during some periods of the day.

### 3.2 Livestock density

**Figure 6:**
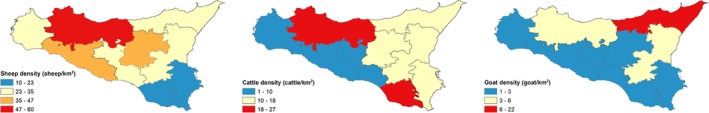
Maps of ruminant livestock density (sheep, cattle and goat) of the Sicilian provinces on 31 August 2017 [Colour figure can be viewed at http://www.wileyonlinelibrary.com]

The density of domestic ruminants susceptible to BTV infection was estimated for all Sicilian provinces on 31 August 2017 (Figure 6), based on data provided by the Italian National Database of the Zootechnical Registry at the ISZAM in Teramo, Italy (http://www.vetinfo.sanita.it). The results indicated densities (animals/km^2^) of 32.57 for sheep, 1.52 for cattle and 1.33 for goats.

## 4 DISCUSSION

This study modelled the feasibility of windborne BTV‐3 introduction from the Cape Bon peninsula in Tunisia to the western Sicilian province of Trapani. Of all the potential routes of introduction to explain Italy's first outbreak of BTV‐3, we focused on aerial introduction of infected *Culicoides* spp. as the most likely. During the study period of 1 August to 18 October, legal movements of animal, semen and embryos from Tunisia to Sicily were not possible, since Council Directive 2004/68/EC did not consider Tunisia a BT‐free region (Council of the European Union, 2004). Illegal movements may have occurred but are less likely. It is possible that infected midges were transported by airplane or ship, especially since Trapani airport lies only 2.3 km from the outbreak, and Trapani port lies 12.5 km from the outbreak. However, no direct flights occurred between Trapani and Tunisia during the study period (Airgest, 2018).

Of the five days when midge deposition was predicted to have occurred in Sicily (2, 10, 11, 12 September; 6 October), the depositions on 2 September were predicted to account for 79.21% (ADS model) or 43.37% (HYSPLIT) of all particles deposited in Trapani during the study period (Table 2). The models give different predictions for deposition on 10 September, which may reflect the different types of deposition stipulated in the two models. Wind speed at ground level was higher on 10 September than on the other days of possible deposition in Trapani (Figure 5); in fact, it was high enough on that day to prevent midge deposition according to the ADS model. Thus, we suggest that 2 September 2017 is the most probable day of BTV‐3 introduction. The fact that the ADS model predicted a deposition location on 2 September that was near, but not identical, to the known outbreak site can be attributed to the spatial resolution of the ADS model for this study. The latitude distance between grid centroids was of 0.1°, translating to an ADS model error of ± 11.1 km. Within this error, the predicted sites of greater midge deposition overlap with the outbreak location.

The prediction of September as the month when infected *Culicoides* spp. were introduced into Trapani correlates with the *Culicoides* spp. population peak in Tunisia (Sghaier et al., 2017). The average temperature in Sicily in September 2017 was 25 ºC, and adult Obsoletus complex and *Culicoides sonorensis* midges can survive for up to three months at, respectively, 17–25ºC and 10ºC (Goffredo, Romeo, Monaco, Di Gennaro, & Savini, 2004; Lysyk & Danyk, 2007), while *C. sonorensis* can live for up to 28 days at 30ºC (Lysyk & Danyk, 2007). Although *C. imicola* lifespan has been less studied, adults can survive more than 15 days (Nevill, 1971; Paweska, Venter, & Mellor, 2002). Given that the outbreak in Sicily involved BTV‐3‐naïve animals and did not lead to multiple outbreaks, we hypothesize that a small number of BTV‐3‐infected midges arrived at one location on the western Sicilian coast and were sufficient to transmit BTV to a susceptible host (Baylis, O'Connell, & Mellor, 2008) in late September or early October.

Among the potential hosts in Trapani, sheep were present at higher density than cattle and goats at the predicted time of BTV‐3 introduction. This may explain why the outbreak occurred in a flock of sheep. Indeed, the ADS model predicted particle deposition on 11 September in Agrigento, which neighbours Trapani to the south and features an even higher sheep density. This may have increased dispersion of the disease.

By April 2018, no other BTV‐3 outbreak had occurred and testing of animals on the affected farm showed them to be negative for BTV‐3 infection (OIE, 2018). Several factors may have helped limit this outbreak, just as they may have helped limit previous outbreaks of BTV‐2 in 2000 (Calistri et al., 2004) and BTV‐1 in 2013 in Sicily (IZSAM G. Caporale, 2013). *Culicoides imicola,* considered the main BTV vector, shows a fragmented distribution on the island (Blanda et al., 2018; Torina, Caracappa, Mellor, Baylis, & Purse, 2004). *Culicoides* spp. are significantly less abundant in November than in October (De Liberato et al., 2010; Goffredo, Conte, & Meiswinkel, 2004). These factors may have strongly limited disease spread, given that only one animal was affected in the 2017 outbreak. We are aware of one other report of a BT outbreak on an island that was potentially due to windborne introduction (Burgin et al., 2013), and that did not spread. In late 2007, a single sheep in a flock on the island of Lolland in Denmark showed BTV‐like clinical signs and tested positive for BTV‐8 infection (OIE, 2007; Rasmussen, Rasmussen, Belsham, Strandbygaard, & Botner, 2010). These two examples of isolated outbreaks that did not spread highlight the importance of environmental factors as well as host and vector densities for BTV propagation.

*Culicoides imicola*'s thermal limits are applied in the ADS model to predict survival rates of *Culicoides* spp. (Fernández‐Carrión et al., 2018), and we set the size of *Culicoides* spp. equal to that of *C. imicola* (Morag, Mullens, & Gottlieb, 2013). We used *C. imicola* as a reference because it is one of the most important vector species in the Mediterranean Basin, and it is the most abundant in the Nabeul Governorate and Tunisia as a whole (Sghaier et al., 2017). While temperature requirements can differ substantially between *C. imicola* and the Palaearctic species (Wilson & Mellor, 2009), the latter appear to be absent from Tunisia or at least present at very low levels (Sghaier et al., 2017). Thus, the model is suitable for the study in this region.

The ADS and HYSPLIT models showed good agreement (Table 2, Figure 4). They predicted the same prevailing winds and differed primarily in how far into Sicily the particles were deposited (Figure 4). These differences can be explained in part by the fact that the ADS model takes into account particle size and density (Fernández‐Carrión et al., 2018), while the HYSPLIT model does not, since velocity is defined for dry deposition (Durr et al., 2017). In addition, both models rely on different meteorological datasets. The HYSPLIT model uses data from the GDAS1, which includes vertical wind velocity, 1‐degree grid resolution and a time window of 3 hr (Stein et al., 2015). Meteorological data in the ADS model lacks some aspects of vertical wind velocity but features spatial resolution of 1 hr (Fernández‐Carrión et al., 2018). We suggest that 1‐hr temporal resolution is sufficient to allow precise real‐time and retrospective analysis of *Culicoides* spp. aerial transport without posing excessive computational demands.

## 5 CONCLUSIONS

Two models suggest that BTV‐3 could have arrived via windborne transport of infected midges to the Trapani province of Sicily, which may explain the occurrence of BT in a single animal there in October 2017. The most likely day of introduction was 2 September 2017. Our results confirm the power of the ADS model for retrospective windborne transportation of* Culicoides* spp. across large water bodies, demonstrating its potential as a surveillance tool for early detection of vectorial diseases and for guiding the early implementation of control measures such as vaccination and bans on susceptible animal movements. Thus, we recommend the ADS model for retrospective and real‐time long‐range insect dispersion studies.

## CONFLICT OF INTERESTS

The authors have no conflict of interest to declare.

## AUTHOR CONTRIBUTIONS

CAV, EFC and JMSV identified the research question and selected the methodology to be used. EFC provided software developments. All authors run the models, contributed to the critical review of the results and approved the final version of the manuscript.
