# Supplementary material for: The possible route of introduction of bluetongue virus serotype 3 into Sicily by windborne transportation of infected Culicoides spp
Source: Transbound Emerg Dis. 2019 Apr 29;66(4):1665–73. doi: 10.1111/tbed.13201 (PMC6850078; doi:10.1111/tbed.13201)
Supplement: Supplementary file 2 [file TBED-66-1665-s002.docx]

**Video S1:** Video of the ADS model simulation for the study period (1 August to 18 October, 2017). The video shows particle movement in the air and ground concentration in the target territory (mp4).
